# The Heart of Medicine

**DOI:** 10.1371/journal.pbio.0030425

**Published:** 2005-12-13

**Authors:** Paul W Glimcher

## Abstract

In *De Motu Cordis,* William Harvey described the circulation of the blood and, in the process, built the first comprehensive biomedical theory on an experimental base.

In the early 16th century the practice of medicine and the discipline of physiology bore almost no relation to the sciences we know today. Medieval scholasticism had entrenched the writings of ancient authors as the arbiters of truth and almost completely suppressed novel inquiry in the biological sciences. The result of this entrenchment was that in European biomedical centers like the Universities of Bologna, Padua, or Montpellier, the writings of ancient medical authors were taught as fact. Direct observations on tissue, and hypothesis-testing experiments of the type routinely performed today, were essentially unknown.

Preeminent among the ancient medical writers for European and Arab scholars was the second century Greek physician Claudius Galen, whose works served as the unquestioned authority on all matters of physiology. Perhaps the most ironic aspect of this cultural devotion was Galen's own rejection of purely scholastic traditions. As he wrote in *On the Natural Faculties:* “The student must learn thoroughly all that has been said by the most illustrious of the ancients. And when he has learnt this, then, for a prolonged period, he must test and prove it, observing what point is in agreement, and what is in disagreement, with obvious fact; thus will he choose this and turn away from that.” Until the sixteenth century, however, the biomedical disciplines rested upon a slavish devotion to the writings of Galen, and his exhortation to perform experiments was ignored.

This Scholastic devotion to Galen was first challenged by Andreas Vesalius, the 16th century father of modern anatomy. Vesalius's brilliant anatomical woodcuts were scrupulously drawn from his own dissections and are probably known today to all students of medicine and physiology. Vesalius's drawings and dissections were specifically meant to challenge the authority of Galen, and his stated goal was an outright overthrow of the hegemony of Scholasticism in the medical academy. What is surprising about Vesalius's work, however, is that it is almost purely descriptive. Nowhere in his work do we see the formal tradition of hypothesis testing by experiment upon which modern biomedical science rests. That tradition begins instead almost a hundred years later in the hands of William Harvey. Perhaps surprisingly, despite the fact that modern biological science is cast almost entirely in the image of Harvey's model, his works are much less familiar than those of Vesalius to modern practitioners of science. With that in mind, it seems particularly timely to review Harvey's master work: *De Motu Cordis*, or *On the Motion of the Heart and Blood in Animals*, a brief and easily read work upon which the bulk of modern medical science and method rests. [Fig pbio-0030425-g001]

**Figure pbio-0030425-g001:**
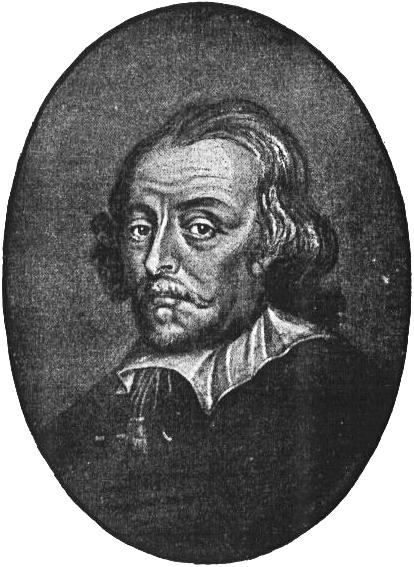
William Harvey (Image: University of Texas Libraries, University of Texas at Austin)

William Harvey was born in England in 1578 into a large and well-to-do merchant family. He received a traditional English education focused on Latin and Greek classics before enrolling in Gonvil-Caius College at Cambridge. After completing a bachelor's degree there at the age of 19, Harvey moved to Padua, Italy, to undertake his medical training under several of the leading physicians of his day, including his personal mentor Fabricius of Aquapendente. After five years at medical school Harvey returned to England and set about building his practice as a physician and conducting a series of private researches.

Of particular interest to Harvey was the mystery of the pulse and its relationship to the beating of the heart. It had been recognized for centuries that the arteries expanded and contracted at regular intervals, and that if a tourniquet was placed on a limb the arteries distal to the tourniquet ceased to pulse and the limb grew cold. Also it was known that all arteries were connected to the left side of the heart and that this organ was connected to the lungs. From this, it was generally concluded that the arteries themselves, by some form of muscular action, expanded and drew either blood or air into themselves from the heart or through the skin. The heart was widely presumed to contract in phase with the arteries. The distinct venous system was believed to mediate the passing of nutrition from the gut to the periphery via the blood. Food was transformed in the gut to chyle, which entered the liver via the portal circulation and then passed to the venous system for distribution throughout the body.

Harvey undertook a series of experiments designed to test this set of assertions, and it is a description of these tests that form the first six chapters of the *De Motu Cordis*. In the *Proeme* (Introduction) of the book, Harvey sets out to demonstrate that this contemporary description of the pulse simply cannot be correct. Immerse a man in a bath of oil, taking his pulse before and after immersion. The strength of his pulse is undiminished by the immersion even though the ability of air to penetrate the skin must in some degree be reduced by this immersion. Section an artery, and you observe not only that blood pulses out of the proximal side of the cut but that no air rushes into the distal side, nor is a pulse observed on the distal side. In short, perform a set of simple demonstrations aimed at testing this standard view and you are driven to the conclusion that it cannot be correct.

In the six chapters that follow Harvey presents a series of descriptive observations that yield an entirely novel conclusion: that the blood is driven into the arteries by a contraction of the left ventricle of the heart and that this blood is supplied to the left ventricle by the right ventricle, from the lungs. This blood, in turn, comes from the vena cava via the right side of the heart, which acts to drive blood through the lungs. To prove this, Harvey presents a series of very compelling observations made under a wide range of conditions. He observes that amphibian or reptilian hearts, which have been chilled to reduce the speed at which they beat, contract at the same time as the aorta to which they are connected fills. He notes that the heart looks like nothing so much as a muscle and that it becomes rigid when contracted. He notes that in the embryo the foramen ovale directly connects the right and left side of the heart, bypassing the lungs and providing a sure and simple passage between the venous and arterial systems. From these he concludes that “the pulsations of the arteries arises from the impulsion of the blood from the left ventricle; just so, as when one blows into a glove, he shall see all the fingers swell up together and assiumulate [sic] this pulsation.”

These first six chapters of the book are without a doubt worth reading, but to my mind what is most striking about them is how unmodern they feel. There is no doubt that Harvey is challenging Galenic doctrine, but he does so with a series of observations that a modern scientist cannot possibly consider experiments. These chapters are good, but not great. Apparently, these chapters reflected a preliminary series of observations that Harvey probably first presented as a series of lectures in anatomy in London and likely wrote up as a very short pamphlet that was never published independently. What probably prevented the publication of that pamphlet was Harvey's recognition that his observations raised more questions than they answered. If blood is pumped by the heart into the arteries, where does it go? If the venous system supplies blood to the heart, from where does this blood come? Harvey must have recognized that his observations yielded a system that was only slightly more logical than the one he was arguing against. It is in a reaction to this puzzle that Harvey's real genius showed itself and invented what we think of today as the biomedical scientific method.

Driven by these observations, in the second half of the book, Harvey sets out to prove a simple hypothesis by quantitative experiment. To us that seems a clear strategy, but in 17th century Europe the concept of experiment was almost unknown and the use of quantitative experimental strategies was only just starting to be used by Galileo in Italy. The first compelling experiment Harvey provides is to measure the output of the left ventricle at each contraction. He then goes on to very conservatively measure the volume of blood pumped by the heart into the arteries with each contraction. Making conservative estimates of cardiac rate, he then computes the total volume of blood moved by the heart in a day. This volume, he concludes, exceeds the weight of a man. In another experiment he measures the volume of blood that passes out of a cut carotid artery and concludes that it accounts for the entire blood volume of an animal in only 15 minutes. [Fig pbio-0030425-g002]

**Figure pbio-0030425-g002:**
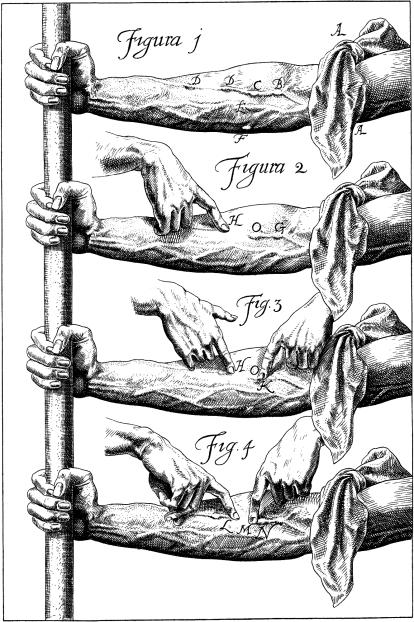
Figure from *On the Motion of the Heart and Blood in Animals* (1628) (Image: National Library of Medicine)

In the second half of the book Harvey presents a number of experiments like this that are absolutely delightful to read. In perhaps the most famous of these experiments he examines the actions of tourniquets and investigates the functions of the valves within veins during the cardiac cycle. In that investigation, which he encourages his reader to attempt, a tourniquet is placed on the upper arm. As the veins distend, small bumps along the veins become visible, which he identified as “little swellings” made by the venous valves, or “portals.” “If you draw down blood with your thumb or finger from [one node to the next] you see that [no blood] can follow … and yet [the vessel is] full enough above the knot…. Hence, since a man may make experiment in many places, it appears that the function of the portal in the veins is the same as that of the [the sigmoid valves of the heart]…to wit that they should be closely shut up, lest they should hinder the blood to return back again.” An experimental passage aimed at proving the unidirectional flow of blood in the veins as required by his hypothesis.

These experiments are all lovely to read, not simply because they invented modern biomedical science but because they provide such an elegant proof of a complex hypothesis. Indeed, Harvey even concludes his experimental sections with a model for the modern discussion section of a scientific paper: “It must of necessity be concluded that the blood is driven into a round by a circular motion in creatures, and that it moves perpetually; and hence does arise the action and function of the heart, which by pulsation it performs; and lastly, that the motion and pulsation of the heart is the only cause.”

By the time Harvey had written the second half of the *De Motu Cordis*, he had risen to the top of British medical practice. At that time he was physician to King Charles I, as he had been to Charles's father, James I. The *De Motu Cordis* was, however, not immediately accepted, and Harvey reported to a later biographer that his medical practice suffered terribly after the book's publication. His life was further complicated by the English civil war, which brought Cromwell to power and cost Charles his head. Harvey was, by all accounts, close to Charles and was deeply wounded by his sovereign's execution. Despite his ties to the crown before the civil war, however, Harvey was lionized towards the end of his life. He was, for example, elected president of the Royal College of Physicians (an office he declined) and widely hailed throughout Europe as the preeminent biomedical scientist of his period. It was clear by the end of Harvey's life what he had accomplished; he not only had solved the puzzle of the blood, the heart, the lungs, and the pulse but had done something much more significant than even that. He had described a method by which hypotheses could be tested by quantitative experiment. Historians often credit Francis Bacon with this accomplishment because he wrote at the same time about how scholars ought to do experiments, but it was Harvey, not Bacon, who built the first comprehensive biomedical theory on an experimental base.

The *De Motu Cordis* is striking because it accomplishes this in about a hundred pages. What I like best about the work, however, is the way it really is two books in one. The first is a not terribly exciting early Enlightenment scientific tract that is well worth reading if you are a historian of science. The second half is a model, or rather *the* model, of experimental hypothesis testing, and it is this half that really soars. For the English-language reader, there are basically two translations of the *De Motu Cordis* from the original Latin available today. The first is an Elizabethan translation produced during Harvey's lifetime and the second is Robert Willis's translation, produced in the mid-1800s. Both have their virtues. Willis's translation is definitely the more readable to a modern biologist, but to my mind, the Elizabethan translation, which sounds almost Shakespearian, captures an excitement that the more modern translation misses. One can only hope, regardless of the translation, that as each generation of biomedical scientists turns towards the history of their field, Harvey will be rediscovered and emulated in the same way Harvey rediscovered the true meaning of Galen.

## Supporting Information

Text S1 On the Motion of the Heart and Blood in Animals (181 KB TXT).

**Box 1**

Harvey W (1628) On the motion of the heart and blood in animals. Willis R, translator. In: Scientific papers (physiology, medicine, surgery, geology). Salt Lake City: Project Gutenberg. Available: http://onlinebooks.library.upenn.edu/webbin/gutbook/lookup?num=5694. Accessed 18 October 2005. Also available as Text S1.

